# MEDTEG (Minimum Entropy Dynamic Test Grids): A Novel Algorithm for Adding New Test Locations to a Perimetric Test Grid

**DOI:** 10.1167/tvst.14.2.25

**Published:** 2025-02-26

**Authors:** Pete R. Jones

**Affiliations:** 1 Department of Optometry and Visual Sciences, School of Health & Medical Sciences, City St George's, University of London, London, UK; 2 UCL Institute of Ophthalmology, London, UK

**Keywords:** perimetry, visual fields, test grid, entropy, dynamic test grids, maximum a posteriori, voronoi tessellation, delauney triangulation, information theory

## Abstract

**Purpose:**

To describe a novel algorithm (MEDTEG) for dynamically adding new test locations to a perimetric grid—to provide a more personalized/comprehensive visual field (VF) assessment.

**Methods:**

MEDTEG operates by finding the most *informative* new test location. First, Voronoi tessellation is used to construct a list of candidate locations (i.e., points that lie in between the current test locations, even when the current grid is sparse or irregular). Next, each candidate's probability mass function is computed using natural neighbor interpolation. Finally, the most informative candidate is determined by computing the expected reduction in entropy (after trial *t* + 1) and then multiplying this value by the area of its Voronoi cell, to estimate the overall volume of expected information gain. Optional weighting coefficients can be applied to encourage/restrict testing to particular spatial locations (e.g., to avoid the midline, target the macula, or prioritize regions exhibiting structural damage).

**Results:**

Using a combination of mathematics, graphics, and MATLAB code, we describe the algorithm and simulate possible use cases. These include ways of providing more detailed evaluations of small scotomas (“enhanced perimetry”), more efficiently assessing patients with extensive loss (“personalized perimetry”), or maximizing VF information in patients with limited attention spans (“indeterminate duration perimetry”).

**Conclusions:**

Simulations of perimetric data indicate that MEDTEG provides a logical and flexible way of automatically adding test locations to an existing perimetric test grid, or of constructing entirely novel grids based on a handful of seed locations.

**Translational Relevance:**

MEDTEG may facilitate more informative VF assessments or allow testing in challenging populations.

## Introduction

### What to Present Next?

In standard automated perimetry (SAP), a key function of the automated algorithm is to determine “what stimulus intensity to present next?” Numerous potential solutions have been proposed^1^ (“advance in –4/+2 dB steps following each correct/incorrect response,”^2^ “present the mean of the posterior mass function,”^3^ and various other heuristics).^4^ Many believe,^5^^–^^7^ however, that as a general rule (i.e., in the absence of any countervailing imperative), one should present the most *informative* stimulus. In technical terms, this can be operationalized as the stimulus that minimizes expected entropy^6^ (i.e., that minimizes threshold estimation uncertainty). This “entropy minimization” approach is simple, flexible, and powerful and lies at the heart of several popular and highly efficient psychophysical algorithms, including QUEST+^8^ and the quick contrast sensitivity function (qCSF).^9^

### Where to Present Next?

The question of “*where* to present next?” is less often considered (though see other studies^10^^–^^12^). This is understandable. Simplicity is important, and so too is standardization (both across patients and for comparison within a patient over time). Using the same fixed test grid to assess every patient on every visit is therefore attractive.

Yet, a one-size-fits-all solution is seldom ideal, and situations exist where assessing alternative and/or additional visual field (VF) locations might be advantageous. A patient may be at floor (no measurable vision) at many of the standard locations, and so benefit from redistributing test points to regions of residual vision (“personalized perimetry”). Or one may want to more precisely delineate a small scotoma (“enhanced perimetry”) (e.g., due to diabetic retinopathy or a localized vascular lesion).

Regardless of the reason why, this article considers how to automatically determine a new VF location to assess. And it shall propose that the answer remains fundamentally the same as when selecting a new stimulus intensity: one should select the most *informative* stimulus. Or, in more technical terms: the test location that minimizes expected entropy.

### Overview of the Present Work

The novel minimum entropy dynamic test grids (MEDTEG) algorithm is detailed in the next section. In brief, this algorithm (1) automatically determines a list of candidate locations and (2) selects the most informative candidate. The sections that follow then briefly describe four simple simulations designed to illustrate possible use cases for the MEDTEG algorithm.

Note that for tractability, we shall restrict ourselves purely to the question of “where to assess next?” and assume that the decision to test a new location has already been made. We shall not consider related questions, such as, “Should we test a new location or continue to test the current location(s)?” although in principle, the present approach could be extended to address such questions also (see Discussion).

## The MEDTEG Algorithm

### Overview

The MEDTEG algorithm is described in more detail below and is available as executable MATLAB code at www.github.com/petejonze/MEDTEG. In brief, MEDTEG proceeds in two stages:

A. **Compile a list of candidate locations**. First, bounded Voronoi tessellation (see below) is used to compile a list of possible test locations (i.e., points that lie in between the current, preexisting test locations, even when the current locations are sparse or irregularly distributed).
B. **Score each candidate.** Second, the most informative candidate location is determined. To achieve this, for each candidate we:
  i. **Compute** ***H***. To compute current entropy, *H*, a predicted probability mass function (PMF) is estimated for each candidate by averaging the PMFs of the candidate's natural neighbors.
  ii. **Compute** ***E***(***ΔH***). The expected reduction in entropy after the next trial, *E*(*ΔH*), is computed for each candidate using a preexisting algorithm such as QUEST+.
  iii. **Compute** ***E***(***ΔHdeg***^**2**^). *E*(*ΔH*) is multiplied by the spatial area of the candidate's Voronoi cell to determine the overall volume of expected information gain.
  iv. **Compute** ***ωE***(***ΔHdeg***^**2**^). By applying optional weighting coefficients, one can encourage/restrict testing to particular spatial locations (e.g., to avoid the midline, target the macula, or prioritize regions exhibiting structural damage).

The best (most informative) candidate is the one that maximizes *ωE*(*ΔHdeg*^2^). This is the location that MEDTEG will recommend assessing on the next trial.

### Compiling a List of Candidates

**Figure 1.**
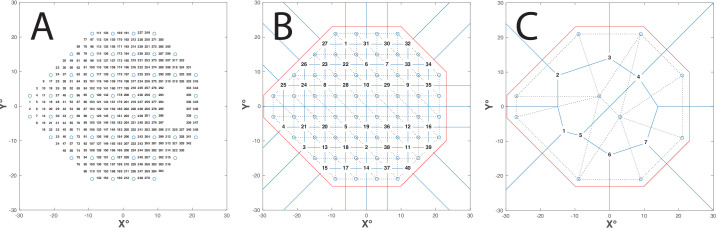
Compiling a list of candidates. (**A**) The naive/manual method applied to a 24-2 grid. Here we simply prespecify candidates interdigitating at 2° intervals. *Blue circles* indicate the previously tested locations. Numbers indicate the possible new candidate locations. Note the large number of candidates required here even for 2 degrees spatial precision (*N* = 348). (**B**) The proposed/automated method applied to a 24-2 grid. Voronoi tessellation (*blue lines*) is used to find all those points lying equidistant to three or more previously tested locations, excluding any points lying outside of their convex hull (*red lines*). Note that since in this example we are starting with a regular grid, and so the candidate locations are also initially equally spaced and predictable. However, after every new test location is added, the grid of possible candidates will become increasingly irregular and unpredictable (hence the need for Voronoi tessellation when compiling a list of possible candidates, even when using a regular starting grid). (**C**) Proposed method applied to arbitrary seed locations. This illustrates how the proposed method can be applied even to sparse or irregular starting grids. Note these images, along with those shown in Figure 2, were generated using the MATLAB code available at www.github.com/petejonze/MEDTEG. This repository also provides a video showing the MEDTEG algorithm running step-by-step.

The first stage of the algorithm is to compile a list of possible new test locations to evaluate (“the candidates”). The prima facie simplest solution would be to manually prespecify all possible candidates in advance. For example, as shown in Figure 1A, if the starting grid is composed of points spaced ±6°, one might specify a second, high-density grid of interdigitating candidates, spaced ±2°.

However, this “manual” approach is arbitrary and brittle (i.e., candidates would need to be respecified for every test grid). It is also somewhat computationally inefficient (i.e., high spatial resolution at any one location would require hundreds or even thousands of possible candidate locations to be specified across the whole VF^13^^,^^14^).

**Figure 2.**
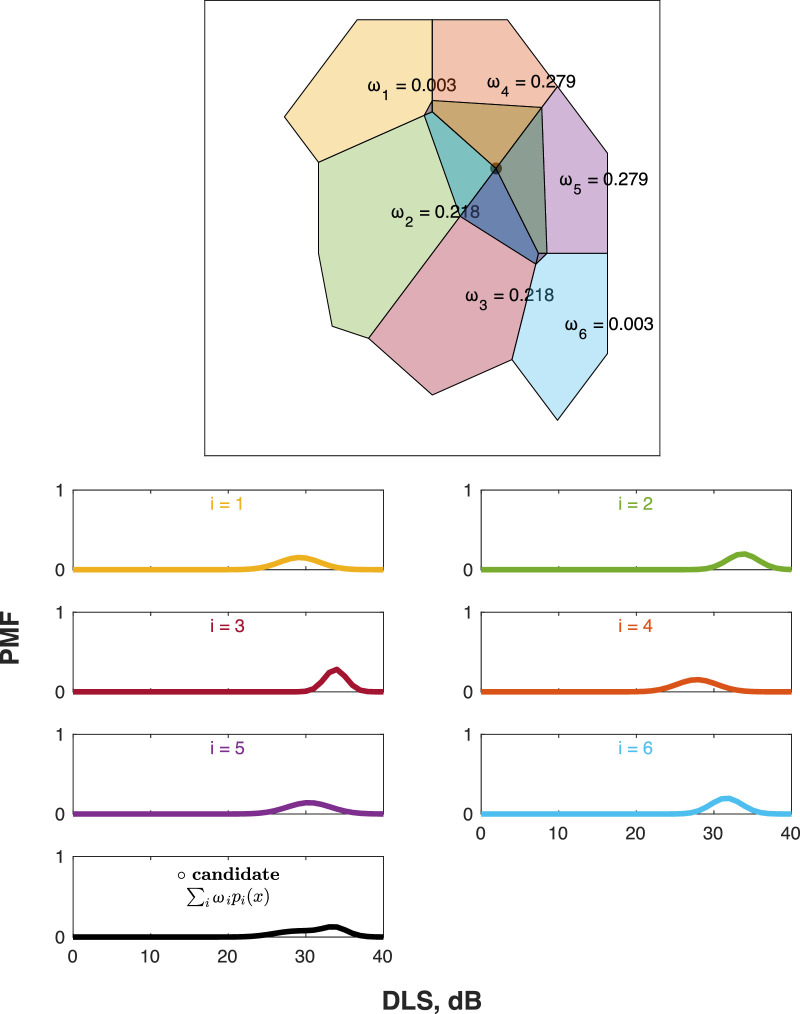
Computing a candidate's PMF. Here we show how a given candidate's PMF is initially predicted using natural-neighbor interpolation. This consists of selecting the candidate's neighbours (in the Voronoi diagram), and averaging their PMFs, with weights proportional to the degree of overlap between the candidate and each neighbor. Thus, in the example shown, the candidate's PMF is strongly determined by test points *i* = 2–5, with minimal input from points *i* = 1 and *i* = 6, where there is minimal overlap (despite these locations not necessarily lying farther away from the candidate). Although not shown here, we could additionally force a weight of zero (*ω* = 0) for any test locations lying on the other side of the horizontal meridian (i.e., to reflect the fact that superior/inferior retinal nerve fibers terminate at the horizontal raphe and do not cross over into the opposing hemifield)^19^ and/or could force *ω* = 0 for points on the other side of the vertical meridian (i.e., in cases of suspected hemianopia).

A different, more flexible approach would be to simply consider as candidates all those locations that lie in between the existing test locations, wherever they lie. As shown in Figure 1B, this can be achieved by using a well-established mathematical technique known as a Voronoi diagram.^15^^,^^16^ In brief, the Voronoi diagram divides the VF into *N* discrete regions (“Voronoi cells”), with each cell representing all those points that lie closer to one test location than any other. In other words, each existing test location becomes the “nucleus” of a Voronoi cell (see Fig. 2 for a graphical illustration). The locations where the boundaries of neighboring cell walls meet (the “Voronoi vertices”) represent points equidistant from three or more of the current test locations. It is these Voronoi vertices that we shall take as our candidate locations (Figs. 1B, 1C, numbers). This approach is as attractive as it is computationally efficient and, as shown in Figure 1C, works even with irregular test grids. As described in “Simulated Examples of Use,” it could even be used to “grow” a fully bespoke grid based on a handful of random or prespecified seed points.

At a later stage of the MEDTEG algorithm, weighting coefficients shall be applied to each candidate to express the user's preference for/against assessing particular VF locations. Attentive readers will note that in the associated MATLAB code, these weights are actually computed now, when compiling the list of candidates. This is for computational expediency since any candidate assigned a weight of 0 will ultimately yield a value of *ωE*(*ΔHdeg*^2^) = 0 and so will never be selected. Such candidates can therefore be discarded immediately without any further evaluation. This is not mathematically necessary, however, and for ease of exposition, we shall postpone describing these weighting coefficients until later (section “Scoring Each Candidate (4 of 4): Computing *ωE*(Δ*Hdeg*^2^)”).

### Scoring Each Candidate (1 of 4): Computing *H*

For each candidate, we begin by computing its current entropy, *H* (i.e., how uncertain we are about the patient's estimated differential light sensitivity [DLS_dB_] threshold at this location). In information theory,^17^ entropy, *H*, is computed from the PMF of an estimated random variable; thus:

(1) $$\begin{array}{c} H=\sum P\left(s\right)\log\left[P(s)\right] \end{array}$$

where *P*(**s**) is the posterior probability of the vector of psychometric function parameters, **s**. Note that where the parameter domain has only one dimension, *H* will be proportional to the variance of the PMF,^18^ with more sharply peaked distributions having lower entropy (i.e., outcome more certain/predictable).

In order to compute *H*, we must first determine the PMF for each candidate. And since, by definition, we have not yet empirically tested the candidate location(s), we shall predict the candidate's PMF by interpolating the PMFs of its neighbors, as illustrated in Figure 2 (i.e., assuming that these have been calculated already due to the routine use of a maximum likelihood algorithm such as Zest or QUEST+; see Discussion).

Formally, the candidate's PMF, *pc*(*x*), is computed as the linear weighted sum of its *n* neighbors. Thus,

(2a) $$\begin{array}{c} p_{c}\left(x\right)=\sum_{i=1}^{n}\omega_{i}\cdot p_{i}\left(x\right), \end{array}$$

where the weights, ω*_i_*, sum to 1 across the candidate's *n* neighbors and are proportional to how much of the candidate's Voronoi cell is “stolen” from each neighbor. Thus,

(2b) $$\begin{array}{c} \omega_{i}=\frac{A_{i}}{A_{c}}, \end{array}$$

where *A_c_* is the total area of the candidate's Voronoi cell (i.e., when the Voronoi tessellation is repeated with this point included), and *A_i_* is the area of intersection between the candidate's Voronoi cell and the Voronoi cell of its *i*th neighbor (i.e., in the original Voronoi diagram, with the candidate not included). In this way, the more the new candidate overlaps with an existing test location, the more that location's DLS_dB_ estimate will determine the candidate's starting prior.

Note that such natural-neighbor interpolation^20^ (aka “Sibsonian interpolation”) is preferable to simpler, distance-based methods of interpolation (e.g., nearest-neighbor interpolation) in that it provides a smooth surface free from any discontinuities, makes no statistical assumptions, and is spatially adaptive: automatically adapting to local variation in data density or spatial arrangement. It has also been specifically shown to perform well when interpolating VF data.^21^ It also poses a negligible computational cost in the present context since the requisite Voronoi tessellation is required later anyway (when determining the area of the new Voronoi cell).

**Figure 3.**
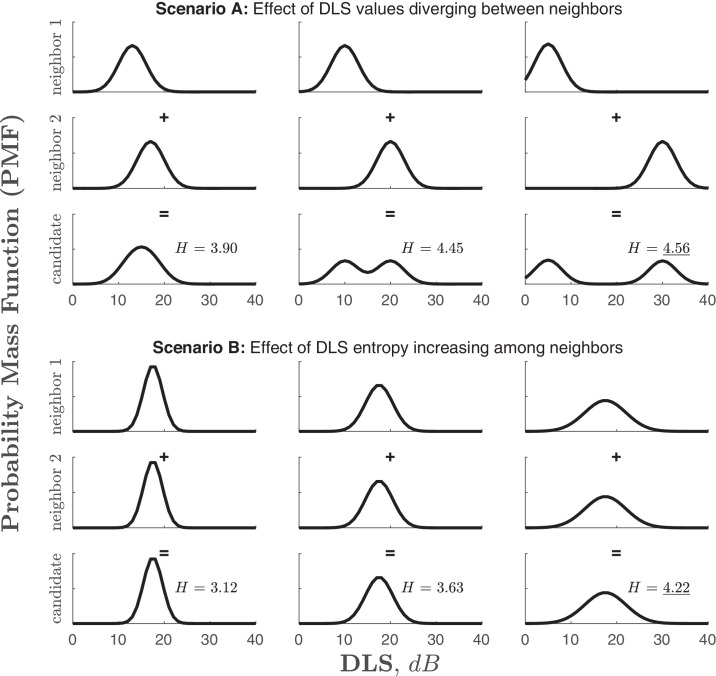
Simplified schematics illustrating how MEDTEG favors testing VF regions with higher measurement uncertainty. In scenario (**A**), we see how predicted entropy (uncertainty) at the candidate increases as the estimated sensitivities of its natural neighbors diverge. In scenario (**B**), we see how predicted entropy increases as the entropy of its natural neighbors increases. In both cases, and if all other considerations are equal (e.g., the slope of the psychometric function), points of high uncertainty, *H*, will be points of high information gain, *E*(*ΔH*) (i.e., *H ∝ E*(*ΔH*)). Thus, the algorithm will be naturally inclined toward testing regions of the VF where visual sensitivity is currently most uncertain, either because sensitivity suddenly changes (from one existing test location to another) and/or because the estimated sensitivity at the existing test locations remains uncertain.

Note that, as shown in Figure 3, when the neighbors’ PMFs are very dissimilar, entropy (uncertainty), *H*, at the candidate location will be relatively high, and so the expected information gain associated with testing this location will also be high too. Conversely, if the neighbors’ PMFs are all similar, then entropy at the candidate location will be low and the expected information gain will be low too. In this way, the algorithm will be naturally inclined toward selecting regions of the VF where DLS_dB_ thresholds change abruptly (e.g., the edges of scotomas). Likewise, as also shown in Figure 3, candidates where the surrounding neighbors have high entropy will be preferred over candidates whose neighbors have low entropy. In this way, the algorithm will be naturally inclined toward selecting regions of the VF where DLS_dB_ thresholds are not easily predicted by the existing test data.

### Scoring Each Candidate (2 of 4): Computing *E*(Δ*H*)

To determine which candidate is likely to be most informative, at each location, we shall compute the expected *change* in entropy after the next trial, *E*(*ΔH*). This can be achieved using any commonly available MAP algorithm (e.g., QUEST+).

Note that since *E*(*ΔH*) is computed by the core MAP algorithm, not by MEDTEG itself, a full exposition of their workings is not provided and can be found elsewhere.^8^ A brief overview is nonetheless provided here, as understanding how *E*(*ΔH*) is computed at each candidate location is key for understanding the subsequent steps that follow.

In short, MAP algorithms require us to specify a prior distribution (expressing our current beliefs about sensitivity in this region of the VF) and a psychometric function (expressing how we expect the probability of a correct response to vary with stimulus magnitude).

For the candidate location's prior, we shall use the PMF already computed in the previous step (using natural-neighbor interpolation).

For the candidate's psychometric function, this “frequency of seeing curve” will have been defined in advance (before testing). In our example MATLAB code, the probability of responding correctly, *p*(correct), is assumed to be determined by a modified cumulative Gaussian function, Ψ, with one variable parameter: *µ* (i.e., the estimated DLS_dB_ value) and three fixed parameters (internal noise, *σ*; lapse rate, *λ*; and guess rate, *γ*). Thus,

(3) $$\begin{array}{ccc} & & \;p\left(correct\right)\\ & & \;=\gamma +\left(1-\gamma -\lambda \right)\left[1-\Psi \left(x;\left\{\mu ,\sigma ,\gamma ,\lambda \right\}\right)\right]. \end{array}$$

**Figure 4.**
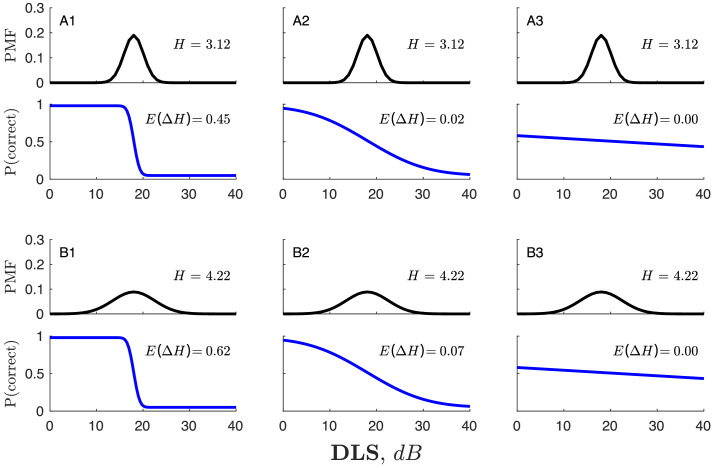
Schematic showing the effect of psychometric slope on expected information gain, *E*(*ΔH*). Panels A1–A3 show the effects of psychometric function slope on expected information gain when current measurement uncertainty (the spread of the PMF) is low. Panels B1–B3 show the effect of psychometric function slope on information gain when current measurement uncertainty is high. The biggest gains in information will exist where both entropy, *H*, and the slope of the psychometric function, *1/σ*, are high (panel B1). Conversely, moving left to right, as the psychometric function becomes flatter, *E*(*ΔH*) decreases. As a result, it may become preferable to test a candidate location about which more is already known (i.e., with a lower entropy, *H*) if the alternatives have higher measurement noise (shallower psychometric functions), as the expected information gain, *E*(*ΔH*), may nonetheless be greater (e.g., compare panel A2 and panel B3).

This psychometric function (illustrated graphically by the blue lines in Fig. 4) is used by the MAP algorithm to compute the likelihood of each possible response given each possible stimulus, and from these values, one can compute the expected change (reduction) in entropy following the next trial, *E(ΔH)*.

Note that the steeper the psychometric slope (and also the lower the values of *λ* and *γ*), the more *informative* the patient's response will be. Conversely, if—*in extremis*—the slope of the psychometric function were to be completely flat (*σ* = ∞), then the probability of responding correctly would always be 50% regardless of the stimulus magnitude. In which case, however the patient responds, we would learn nothing new about what they can or cannot see. In this way, as shown in Figure 4, the algorithm will be naturally inclined toward selecting regions of the VF where the response will be more informative (i.e., where *σ* is lower).

Note also that if (as is sometimes the case) the psychometric function is assumed to be constant across the VF, then this step of the MEDTEG algorithm could be skipped. Thus, instead of computing the expected change in entropy, *E*(*ΔH*), one could simply compute predicted entropy, *H*, and select the region about which we are currently most uncertain (i.e., “*H* max” rather than “*E*(*ΔH*) max”). An *H* max approach would be conceptually and computationally simpler but would be unable to take into account changes in response reliability (i.e., as a function of eccentricity and/or mean sensitivity). Thus, if there were regions of the VF where the psychometric slope was very flat, then an *H* max variant of MEDTEG is liable to get stuck there, repeatedly testing locations where entropy, *H*, is high but where the gain in information, *ΔH*, is low.

### Scoring Each Candidate (3 of 4): Computing *E*(*ΔHdeg*^2^)

Next, we multiply the expected information gain, *E*(*ΔH*), by the area of the candidate's Voronoi cell, *A*_c_, in order to take into account the total *volume* of information gained across the VF:

(4) $$\begin{array}{c} E\left(\Delta H\deg^{2}\right)=E\left(\Delta H\right)\cdot A_{c}. \end{array}$$

**Figure 5.**
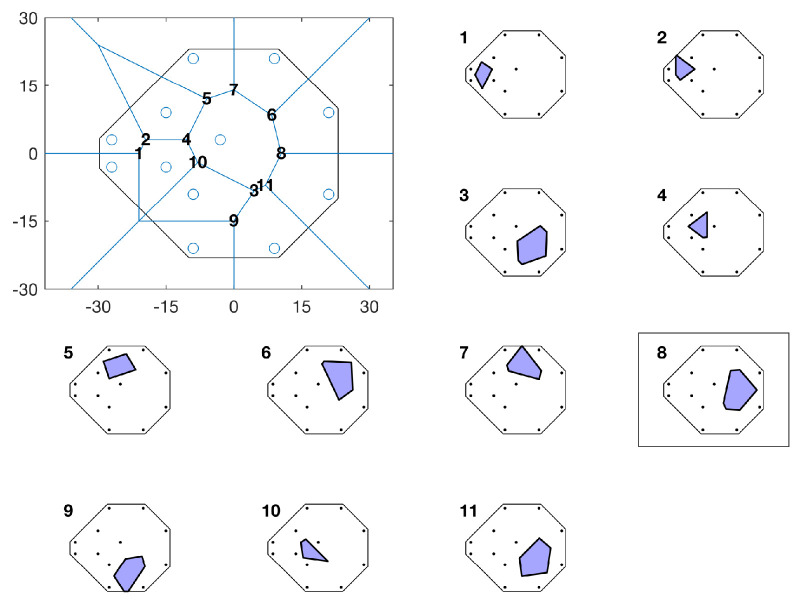
Schematic showing how, all other factors being equal, the algorithm will prefer the candidate that will be informative about the greatest VF extent (in this instance, candidate #8). The *top-left panel* shows points previously tested (*blue circles*) and the 11 new candidates (*numbers*), plotted as a function of degrees visual angle. Each of the subpanels shows the associated Voronoi cell (*shaded polygon*) for the 11 candidates. Given their small Voronoi cell size, a candidate such as #1 or #10 would only be selected, for example, if the expected gain in information, *E*(*ΔH*), was disproportionately large (i.e., if these points had very low measurement error/very high psychometric slopes). Or, alternatively, if these points were assigned higher weights, *ω*.

In this way, as shown in Figure 5, MEDTEG will prefer candidates that are informative about greater expanses of the visual field and will be discouraged from testing regions close to where existing test locations already lie. It will not, therefore, simply cluster points ever closer to the edge of the deepest scotoma, in contrast to a simpler “maximum gradient” algorithm (see section “Simulated Use Case 2”).

### Scoring Each Candidate (4 of 4): Computing *ωE*(*ΔHdeg*^2^)

Finally, a scalar weight, *ω*, is applied to each candidate to express any a priori preferences for/against assessing particular VF locations. Thus,

(5) $$\begin{array}{c} \omega E\left(\Delta Hdeg^{2}\right)=E\left(\Delta Hdeg^{2}\right)\cdot \omega , \end{array}$$

where *ω* is a user-specified value between 0 and 1 (0 ≤ *ω* ≤ 1). Note that these (“per-candidate”) weights are unrelated to the (“per-neighbor”) weights in Equation 2a (used when interpolating neighboring PMFs).

**Figure 6.**
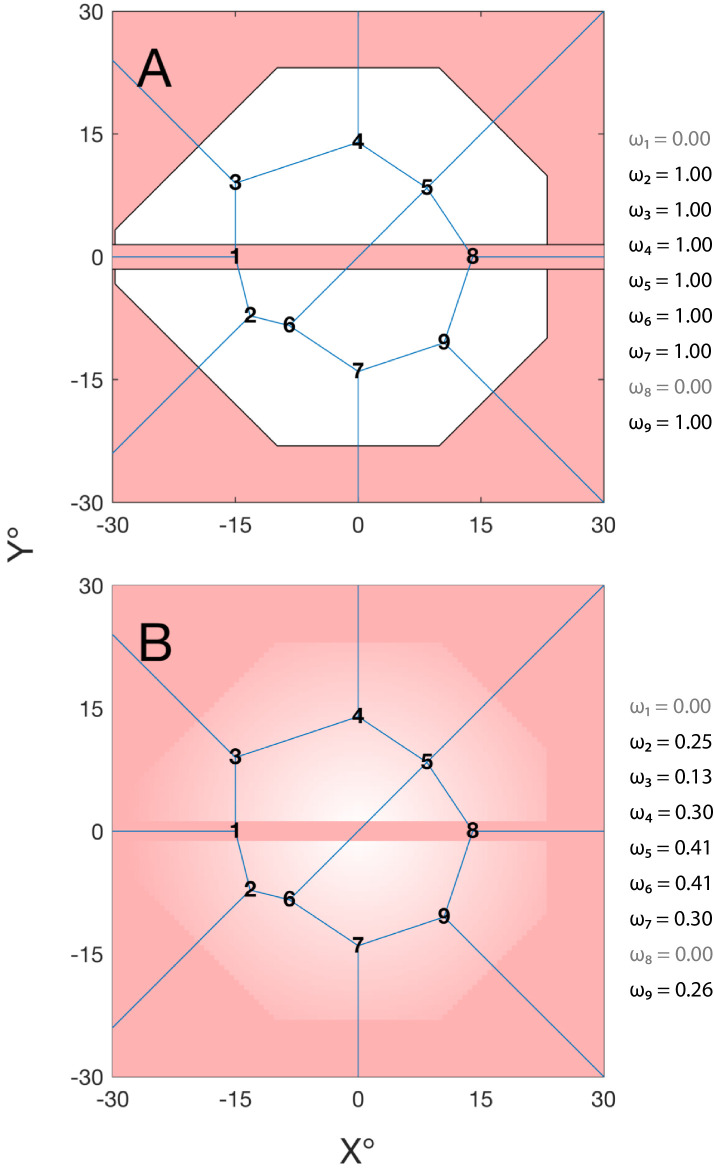
Schematic showing how the user can specify weights, *ω*, to candidates based on their location (*white* = high weight; *red* = low weight). This can be done to encourage selected test locations to fall within certain parts of the VF or to preclude testing in certain regions. For example, in (**A**), new candidates are required to fall within the convex hull of the standard 24-2 test grid and are not permitted to fall within 1° of the horizontal meridian. Candidates that fail to meet these criteria (i.e., that fall in the *red regions*) are assigned a weight of 0 and so never selected. In (**B**), these binary weights are further supplemented with gradated values that decrease with eccentricity to favor more central candidate locations (*ω* inversely proportional to the distance from fixation). Note, however, that a more eccentric location may still ultimately be selected if, for example, the expected gain in information, *E*(*ΔH*), is much larger and/or the spatial extent of its Voronoi cell, *A_c_*, much greater.

As shown in Figure 6A, all users will likely want to set *ω* = 0 for any candidates that fall outside the testable spatial range of the device, and most users (particularly those interested in glaucoma) will want to set *ω* = 0 for locations lying close (e.g., <1°) to the horizontal meridian^19^ or that fall within the physiologic blind spot. In addition, some users may want to set *ω* = 0 for any points that fall outside the convex hull of the original points (i.e., to constrain testing to locations inside the existing grid and not increase its spatial extent). Finally, more nuanced weights can also be employed. For example, *ω* could be set to decrease gradually as a function of eccentricity to prioritize testing of central regions, as shown in Figure 6B.

In this way, the algorithm can be made to prefer candidates that lie in regions of interest, howsoever the user wishes to define such regions (i.e., based on a priori anatomic considerations, structural data, or the importance of certain parts of the VF to the patient or clinician).

### Selecting the Best Candidate

Selecting the best (most informative) candidate, *x*, is generally a case of simply iterating through the whole set of possible candidates, *S*, and selecting the candidate with the greatest *ωE*(*ΔHdeg*^2^) value:

(6) $$\begin{array}{c} x=\arg\max_{x\in S}\left[\omega E\left(\Delta Hdeg^{2}\right)\right]. \end{array}$$

Alternatively, one could select the top *N* candidates if wanting to add new locations in batches, or one could randomly select candidates with weights proportional to* ωE*(*ΔHdeg*^2^), if wanting to introduce a degree of stochasticity. As with all good psychophysical algorithms (e.g., QUEST+), on any given trial, the user is free to override MEDTEG's recommendation and manually select any test location they choose (e.g., if one wanted to insist on a certain, fixed test location at the start of the test for practice/familiarization purposes).

### Key Qualities of the MEDTEG Algorithm

The MEDTEG algorithm has several attractive properties. First, it is flexible, in that it can be applied to any arbitrary test grid (including irregularly spaced grids) and/or can be applied recursively to the same grid (e.g., to progressively grow the grid dynamically). Second, it is entirely automated, and though its behavior can be customized/constrained by custom parameters, the user is not required to preselect possible candidate locations or is tied to a specific starting grid. This may be particularly attractive to researchers developing standardized protocols (e.g., for clinical trials), particularly when the region of interest is heterogeneous across eyes and/or where test grids are not strongly prescribed (e.g., microperimetry). Third, MEDTEG is capable of automatically balancing multiple competing interests. Thus, as detailed above, it will attempt to select the location where measurement uncertainty is greatest, where the patient's response will be most informative, where no nearby region has been tested previously, and all in accordance with the user's a priori preferences.

## Simulated Examples of Use

Here we describe four simulated use cases. These are designed to illustrate potential applications of the MEDTEG algorithm, but not to formally quantify utility. In each case, SAP threshold estimation and stimulus magnitude selection (dB level) were performed using QUEST+. The probability of the simulated observer responding correctly to a given stimulus dB level was determined stochastically, using the frequency of seeing curve expressed in Equation 2b. The lapse rate, *λ*, and guess rate, *γ*, parameters were fixed at *λ* = 0.05 and *γ* = 0.02*.* The slope, *σ*, and true DLS_dB_ sensitivity, *µ*, varied across the visual field and formed the simulated observer's true “hill of vision.” By default, these values (*σ* and *µ*) were randomly jittered versions of the expected normative values for a normally sighted young adult.^22^ The hill of vision was then modified to include various patterns of VF loss, as detailed in each simulation. For further technical specifics, see https://github.com/petejonze/MEDTEG for the MATLAB code used to run these simulations.

### Simulation Use Case 1: Better Characterizing a Small, Localized Scotoma (“Enhanced Perimetry”)

**Figure 7.**
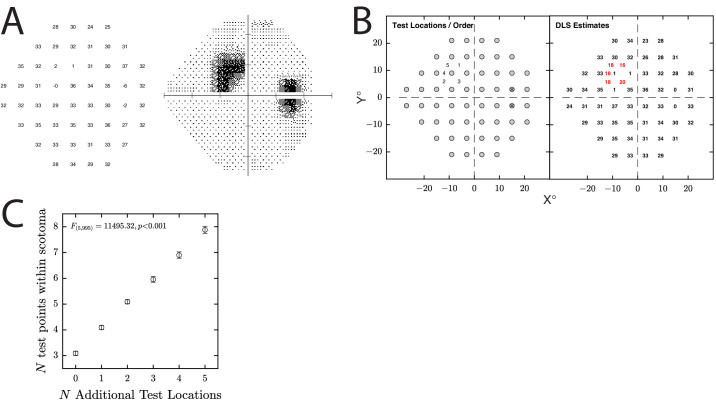
Simulated use case 1: enhanced perimetry. Here we illustrate MEDTEG’s ability to automatically “follow up” on small regions of apparent loss. (**A**) Example simulated observer with three contiguous locations of central loss on the 24-2 grid. Numbers indicate DLS_dB_ values (i.e., the *µ* parameter of the frequency of seeing curve given in Equation 3), and the exact values were randomly jittered on every run. (**B**) Output from a single run of the simulation, showing where the five new test points were placed (*left*) and the resultant DLS_dB_ estimates (*right*). (**C**) Summary of *n* = 200 runs, showing how many of the new points were located in or around the scotoma (the scotoma, in this case, being arbitrarily defined as any location where the simulated loss was at least −8 dB, relative to a normal). Note these simulations, along with the others shown in Figures 8 to 10, were run using the MATLAB code available at https://github.com/petejonze/MEDTEG.

Imagine one is assessing a new patient with optic neuritis using a 24-2 grid. The VF is largely normal. However, three adjacent points in the macula show an apparent scotoma that is cause for concern (Fig. 7A). A clinician wishing to further assess this central region of loss could perform a follow-up 10-2 test. However, this requires an additional *n* = 68 test points, of which only around *n* ≈ 15 points (∼25%) would actually fall in the impaired region.

In contrast, if a human operator were manually controlling the process, after performing the initial 24-2 test, they would solely follow up the damaged region and place any new test points in or around the estimated region of loss. Is MEDTEG able to mimic this more efficient, human behavior?

In simulation 1 (Fig. 7), we first simulated responses at the *n* = 54 standard locations on the 24-2 grid. We then tested five additional points (sequentially) at locations suggested by MEDTEG. As illustrated in Figure 7B and 7C, these additional five points were almost always placed in or around the scotoma, as desired.

### Simulated Use Case 2: More Efficiently Assessing a Patient With Extensive Loss in One Hemifield (“Personalized Perimetry”)

Imagine one is assessing a new neurology patient with a complete loss of sensitivity in one hemifield. Performing a standard 24-2 test would be inefficient since half of the test points would fall in a region of obvious loss. This wastes valuable clinic time, and the patient is prone to become confused or demoralized as they will spend the majority of the test not seeing any targets.

In contrast, a human, once they have established the hemifield loss, would likely spend longer testing in or around the preserved hemifield (i.e., rather than repeatedly probing regions with no measurable sensitivity). Thus, while the optimal test strategy is undefined, it seems uncontentious to suggest that, over time, more test locations should fall in the preserved hemifield than in the nonseeing hemifield.

**Figure 8.**
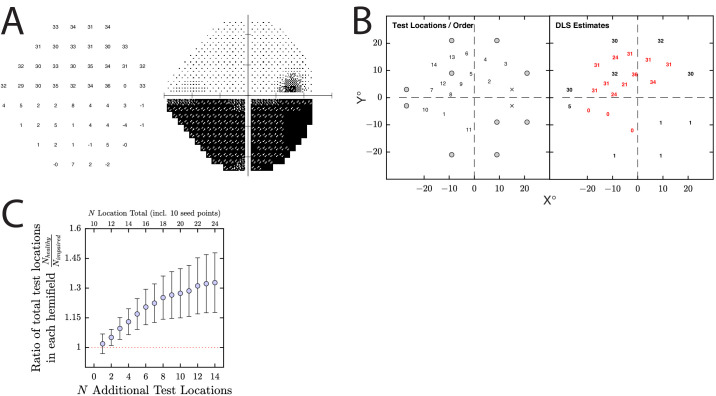
Simulated use case 2: personalized perimetry (figure presented in the same format as Fig. 7). (**A**) Example simulated observer. (**B**) Example result from a single run of the simulation. *Left*: The numbers indicate the order in which the dynamic points were tested, following the initial 10 seed points (*gray* *circles*). *Right:* The DLS_dB_ estimates for each location, with the number in *red* indicating the new locations suggested by MEDTEG. (**C**) Summary of results from all *n* = 200 runs, indicating the proportion of the total test points (including the 10 initial seed points) located in the preserved hemifield (bigger values = better). The *horizontal dashed line* indicates the situation when the number of test points in both hemifields is equal (i.e., as per a standard 24-2 grid). Note that here we simulated an upper/lower hemifield loss, but the same basic principles would apply to a left/right hemianopia (e.g., if assessing neurology patients).

To see whether MEDTEG can also exhibit this behavior, in simulation 2 (Fig. 8), we first tested just 10 prespecified “seed” points (Fig. 8B, gray circles) and then sequentially tested an additional 12 points at locations selected by MEDTEG. As shown in Figure 8C, while MEDTEG placed test points in both hemifields, it exhibited a clear preference toward the preserved hemifield, placing around 50% more. Again, performing as desired.

### Simulated Use Case 3: Integrating A Priori Structural Data into the VF Assessment (“Structure Guided Perimetry”)

**Figure 9.**
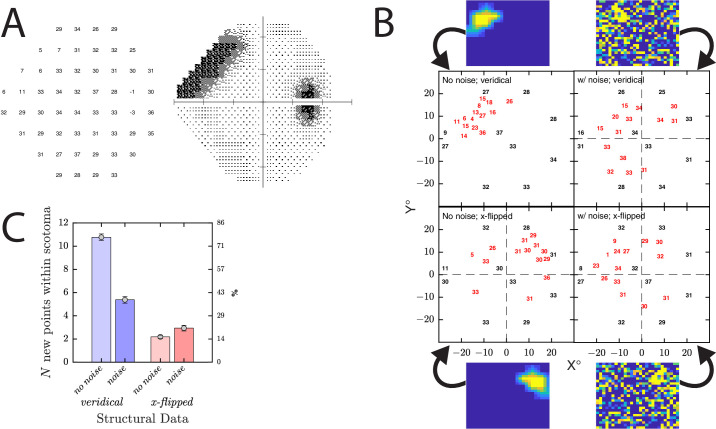
Simulated use case 3: structure guided perimetry. (**A**) Example simulated observer. (**B**) Example result from a single run of the simulation. Numbers indicate the estimated DLS_dB_ values for the initial 10 seed points (*black*) and 14 subsequent dynamic points (*red*) presented at locations determined by MEDTEG. Heatmaps indicate the structural weights, which varied from 0.01 to 1.00 (*blue*: *w* ≈ 0.01; *yellow*: *w* = 1.0). In the noise conditions, each weight value, *w*, was jittered by a random value drawn from a Gaussian distribution, *N*(*µ* = 0, *σ* = 1), and then clamped to the range 0.01 to 1. (**C**) Summary of results from all *n* = 200 runs, indicating how many of the *n* = 14 new points were placed within the damaged (*upper left*) part of the VF (i.e., in regions exhibiting a DLS_dB_ loss of 8 dB or more). *Error bars* correspond to mean ± 95% confidence interval (CI) values.

Exactly how “structure” and “function” are related in perimetry is a large and complex topic^23^ outside the scope of this article. However, simulation 3 (Fig. 9) was intended to illustrate MEDTEG’s ability-in-principle to take into account a priori structural information when deciding which location to test next (i.e., via the weighting coefficients in Equation 5).

Thus, imagine one is assessing a patient with diabetic retinopathy and you have prior warning (e.g., from optical coherence tomography (OCT)) of structural loss in one particular part of the retina. By assigning weights, *ω*, to each candidate proportional to apparent structural dysfunction, we could prioritize perimetric testing in affected parts of the VF (as illustrated by the heatmaps in Fig. 9B).

To illustrate MEDTEG’s capabilities in this regard, in simulation 3, we first tested 10 predefined seed points and then sequentially added *n* = 14 new locations (an arbitrary number), using weights to encourage testing within a region of structural loss. Unsurprisingly, when the structural weights were strong, unambiguous, and corresponded correctly to the area of functional loss, most new test locations (>75%) tended to cluster tightly in the scotomatous region indicated by the structural data (Fig. 9, no noise, veridical).

This behavior is largely trivial and provided only as a sanity check. The more interesting question is how MEDTEG performs if the structural data are noisy or wrong. In those circumstances, a crude algorithm that slavishly followed the structural data would behave erratically and/or fail to ever assess the VF regions containing the true loss of vision. Would MEDTEG behave likewise?

When significant noise was added to structural data, in the form of clamped Gaussian jitter (Fig. 9, noise, veridical), MEDTEG still placed around half of the new points in the region where the scotoma lay, evidencing a degree of robustness to noise. Even when the structural data were flipped horizontally (e.g., due to human error and/or confusion about which eye was being assessed), some points—albeit a minority (∼20%)—were still nevertheless placed within the actual scotoma (Fig. 9, noise, x-flipped), evidencing a degree of robustness to systematic error. Taken together, these results show that MEDTEG is able to integrate structural information, and even when the structural data are wrong or misleading, it is able to perform in a reasonably intelligent manner.

### Simulated Use Case 4: Assessing a Patient With an Indeterminate Attention Span (“Indeterminate Duration Perimetry”)

Imagine one is assessing a young patient with pediatric glaucoma and a limited attention span, the precise duration of which cannot be predicted in advance. A human would keep testing until the child becomes inattentive and then immediately stop, thereby maximizing good data while minimizing bad data. In contrast, automated SAP has historically relied on a prespecified number of test locations. For children, this “one-size-fits-all” approach means either an excessively short test or a longer test that produces catastrophic measurement error in an unknown proportion of cases. Since these bad tests cannot always be easily identified/excluded, all VF tests also become suspect, making SAP in young children largely unfeasible.

Instead, let us make the (nontrivial) assumptions that (1) adherence is binary (either child is paying attention or not), and (2) we have a reliable way of knowing when the child becomes inattentive (e.g., a technician presses a button or some form of automated sensor).^24^^,^^25^ The question then becomes, Can we make more accurate VF assessments by using MEDTEG to prioritize testing the most informative information during the time available? Or is it equally effective just to randomly select points from a standard (e.g., 24-2 grid)?

**Figure 10.**
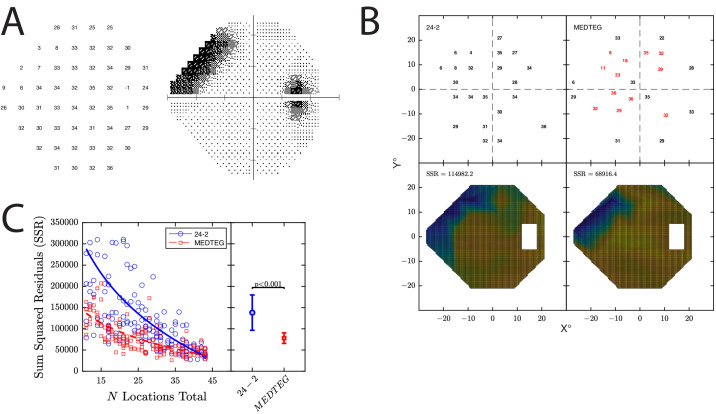
Simulated use case 4: indeterminate duration perimetry. (**A**) Example simulated observer. (**B**) Example result from a single run of the simulation. Numbers indicate the estimated DLS_dB_ values. Heatmaps indicate the “hill of vision” surface, fitted to these DLS_dB_ values using linear interpolation (and excluding points lying in or around the physiologic blind spot). The numbers indicate the sum of the squared residuals (i.e., the residuals being the difference between the predicted and true DLS_dB_ value, evaluated at 0.5° intervals across the VF). Larger SSR values indicate less accurate hill of vision estimates. (**C**) Summary of results from all *n* = 200 runs, indicating SSR values as a function of the number of locations tested (which varied randomly from 11 to 54). Each marker indicates a single run of the simulation. Lines indicate best-fitting logarithmic curves of the following form: *y* = −*a* · *log*_10_(*x*) + *b*. *Error bars* indicate mean ± 95% CI values across all runs, shown with the associated *P* value from a two-sample *t*-test.

To answer this, in simulation 4 (Fig. 10), each test was terminated after a random number of test locations had been assessed (to crudely simulate a sudden loss of concentration). In half of the simulations, the preceding test locations were drawn randomly from a standard 24-2 grid. In the other half, MEDTEG decided where to place each test point in turn (after an initial 10 seed points placed at fixed and largely arbitrary locations). In both cases, the accuracy of the overall VF assessment was evaluated by fitting a surface to the final DLS_dB_ estimates and then computing how closely this fitted surface (Fig. 10B) matched the simulated observer's true hill of vision (Fig. 10A).

As shown in Figure 10C, when the number of locations was large (i.e., a highly compliant patient), the algorithm was immaterial: there was no meaningful difference between the accuracy of VF estimated by the two methods. However, when the number of locations was small (i.e., a patient with a short attention span), the VF tended to be more accurately delineated by using MEDTEG to prioritize the testing of more informative locations.

### A Note on Simpler, Maximum Gradient Selection Strategies

With respect to the preceding four use cases, could similar results be obtained—with less computational overhead—using a simpler algorithm? For example, could one not simply select the candidate location with the maximum difference in estimated threshold between any two existing locations^26^^,^^27^ (i.e., maximizing threshold gradient in Equation 6, rather than the expected change in entropy)?

Example simulations using this simpler “maximum gradient” strategy are shown in Supplementary Figure S1 and demonstrate that the two algorithms sometimes agree but sometimes exhibit qualitatively distinct behaviors.

For use case 1 (*better characterizing a small, localized scotoma*), the two algorithms do behave in a similar manner, with both clustering new, additional test points around the small scotoma (Supplementary Fig. S1A vs. Fig. 7B). Their behaviors would, however, start to diverge as the number of additional test points increases. Thus, the maximum gradient algorithm will always favor the edge of the deepest scotoma. In contrast, while MEDTEG's preference for high uncertainty favors a scotoma's edge, MEDTEG also values locations that are informative about larger spatial extents. The latter preference will cause MEDTEG to increasingly test other parts of the VF as the scotoma's edge becomes congested with test points. Note also that, in the way that they were implemented here, there was also little difference in computational overhead between the two algorithms, since both used Voronoi tessellation to identify the list of candidates, and Sibson weighting of neighboring probability mass functions to initialize the selected candidate (see Discussion for more on computational considerations).

For use case 2 (*more efficiently assessing a patient with extensive loss in one hemifield*), the maximum gradient algorithm tended to only ever place new test points along the meridian separating the preserved/affected hemifields and was as likely to place new points within the affected hemifield as within the preserved hemifield. In contrast, as described previously, MEDTEG tends to place points in the preserved hemifield and attempts to distribute them more widely (Supplementary Fig. S1B vs. Fig. 8B).

For use case 3 (*integrating a priori structural data into the VF assessment*), without the information-theoretic framework provided by MEDTEG, the maximum gradient algorithm lacks any obvious way to integrate prior information into the candidate selection decision. No meaningful comparison is therefore possible.

For use case 4 (*assessing a patient with an indeterminate attention span*), the maximum gradient algorithm tends to tightly cluster test points around the deepest scotoma, with little investigation of the rest of the VF. In contrast, MEDTEG attempted to distribute points across the VF but also exhibited a preference for the region of the VF exhibiting a dense scotoma (Supplementary Fig. S1D vs. Fig. 10B).

## Discussion

This article describes a novel algorithm for dynamically adding new test locations to a perimetric test grid (MEDTEG). Such an algorithm could be used in various ways: to add additional test points to standard test grids (simulated use case 1); replace standard grids with entirely dynamic/bespoke ones, grown from a handful of seed points (simulated use case 2); or use an intermediate approach, where candidates are partially or completely constrained to follow standardized (e.g., 24-2) locations but where MEDTEG's information-theoretic framework is used to integrate a priori structural information (simulated use case 3), or prioritize the most informative locations (simulated use case 4).

### Limitations of the Present Study

Depending on how MEDTEG were to be employed, further careful thought would be required. If grids were made fully dynamic, then conventional summary statistics (e.g., mean deviation, visual field index) would no longer be computable. This, in turn, would complicate many trend-based progression algorithms that rely on established VF metrics. (And even eventbased analyses may become problematic if the number of test locations is no longer constant.) While in more practical terms, for dynamic test grids to be used routinely, clear guidelines would be required (i.e., precisely when/where/how) and protocols developed for how such VF data are stored, visualized, or shared.

Such concerns could in principle be addressed (e.g., by using grid-invariant summary measures, such as volume under the hill of vision). However, it is perhaps more plausible that an algorithm such as MEDTEG would be used to augment rather than replace existing practice (e.g., by adding additional test points to standard grids) or in domains where there are not already well-established norms and practices (e.g., microperimetry and/or fields outside of glaucoma, such as neurology or pediatrics).

Another limitation of the present study is that it does not provide strong, quantitative evidence of MEDTEG's benefits/limitations versus conventional static grids. The simulations provided are only intended as illustrative, and for tractability, various simplifying assumptions were made (e.g., that DLS variance, σ^2^, does not vary with sensitivity, *µ*, which we know to be false,^28^ or that the observer's psychometric function remains stationary throughout the test—also likely false).^29^ Even if the simulations were made more complex, however, they still would not evidence real-world utility. Instead, any truly meaningful test would require a prospective study in real patients. At which point, additional care and consideration would also have to be given to the vagaries of human observers. For example, while in the simulations, individual VF locations were tested sequentially, in humans, any new locations would likely need to be added in batches to avoid the test becoming too predictable. Relatedly, many of the parameters used in the simulations were arbitrary and could be optimized (e.g., the exact number and location of seed points). In general, no other values were ever attempted (both for ease and to avoid “overfitting”), and it is likely that other values would yield more accurate and/or reliable VF assessments.

### Theoretical Limitations of the MEDTEG Algorithm

MEDTEG makes several simplifying assumptions. When considering how informative each potential candidate is, MEDTEG only considers the spatial geometry of the VF (i.e., we assume that a bigger Voronoi cell is always better; see Fig. 5). No consideration is given to the underlying structure of the retina and the fact that, for example, certain parts of the VF may be more clinically useful to assess than others.^30^^,^^31^ Similarly, MEDTEG assumes that the observer's vision is constant within each Voronoi cell (i.e., that their true DLS is flat within the candidate region), which we know is not true. These are limitations that MEDTEG could be modified in future to address. Furthermore, as described in “Scoring Each Candidate (1 of 4): Computing *H*,” MEDTEG also requires various assumptions to be made regarding the shape of the observer's “frequency of seeing curve.” These such assumptions, however, will generally have been made already by the core psychophysical algorithm, independent of whether MEDTEG is used.

More generally, MEDTEG is designed only to answer questions of the form “where,” not “when.” For it to be deployed effectively, one would also have to develop additional algorithms to determine whether or not to test a new location, when to stop adding new points, and/or when to stop the assessment altogether. These “when” questions might also be answered by further generalizing the information-theoretic (“entropy minimization”) approach proposed in this article, just as MEDTEG itself is a generalization of algorithms such as QUEST+ that determine “what” to present. For example, one could employ some form of global VF entropy criterion to determine when to stop testing the whole visual field (just as one might currently employ to determine when to stop testing at a single, given location). Alternatively, one could develop some simple heuristics (e.g., a rule whereby the presence of an apparent central defect triggers an additional *N* test locations). Such considerations lie outside of the scope of the present article, however.

### Practical Limitations of the MEDTEG Algorithm

One key practical consideration is that the MEDTEG algorithm requires access to the raw probability (PMF) information at each preexisting test location, not simply the point estimates typically reported in the final report (i.e., it needs to know the likelihood of each possible DLS_dB_ value, not just what the most likely DLS_dB_ value is). Such information can be computed at runtime by the SAP software from the trial-by-trial response data but cannot be reconstructed retrospectively from a typical perimetric printout. As such (and unlike simpler gradient maximization algorithms), MEDTEG cannot be retrofitted to old data. Instead, MEDTEG is primarily intended for users of perimetric control packages such as the Open Perimetry Interface^32^ (OPI) or for manufacturers of perimeters.

MEDTEG also requires nontrivial computing power (e.g., since, like QUEST+, it uses a brute-force approach to evaluate every possible response, to every possible stimulus, at every possible test location—albeit only looking ahead by a single trial, since beyond that, any additional benefits appear marginal at best).^5^^,^^33^ So long as the number and range of parameters are kept reasonably constrained (e.g., 1 dB stimulus spacing, not 0.01 dB), the search for a new test location can often be completed on the order of tens or hundreds of milliseconds (as evaluated on a MacBook Pro 2023 laptop, with Apple M2 Max CPU). However, if the number of possible test locations is large (i.e., if the existing grid is dense), then it may take a second or more for MEDTEG to determine a new test location. At a minimum, MEDTEG therefore demands modern hardware and sensible programming practices (e.g., asynchronous coding) to absorb this computational period into the pacing of the test without any obvious lag or jitter. If this proved insufficient, then significant speed gains could be achieved by optimizing key components (e.g., porting some or all of the code to native C libraries). While in extremis, the MEDTEG algorithm itself could be modified (e.g., by only evaluating candidates above a certain weight threshold) or key components redesigned (e.g., developing an algorithm to only reevaluate the Voronoi diagram locally, around where new points are inserted, rather than recomputing the whole diagram every time).

## Conclusions and Future Work

We have described a novel algorithm (MEDTEG) for selecting new perimetric test locations using a maximum information gain criterion. We have also demonstrated, by simulation, possible clinical applications, including ways of performing more detailed mapping of scotomas^34^ (“enhanced perimetry”), more efficiently assessing patients with extensive loss in one hemifield (“personalized perimetry”), or maximizing the VF information acquired in a patient with a limited attention span (“indeterminate duration perimetry”).

Code for implementing MEDTEG and also the simulations described is available at www.github.com/petejonze/MEDTEG. In future, it would be particularly desirable to translate this code to R, as an OPI plug-in (https://opi.lei.org.au/). This would allow MEDTEG to be used in conjunction with well-established perimeters such as the Octopus, Heidelberg Edge, IMO, or CenterVue Compass (i.e., pending any MATLAB OPI implementation). We would warmly encourage anybody interested in developing such an R package to fork/modify the GitHub repository.

## Supplementary Material

Supplement 1
