# Supplementary material for: MEDTEG (Minimum Entropy Dynamic Test Grids): A Novel Algorithm for Adding New Test Locations to a Perimetric Test Grid
Source: Transl Vis Sci Technol. 2025 Feb 26;14(2):25. doi: 10.1167/tvst.14.2.25 (PMC11881782; doi:10.1167/tvst.14.2.25)
Supplement: Supplement 1 [file tvst-14-2-25_s001.pdf]

SUPPLEMENTAL MATERIAL

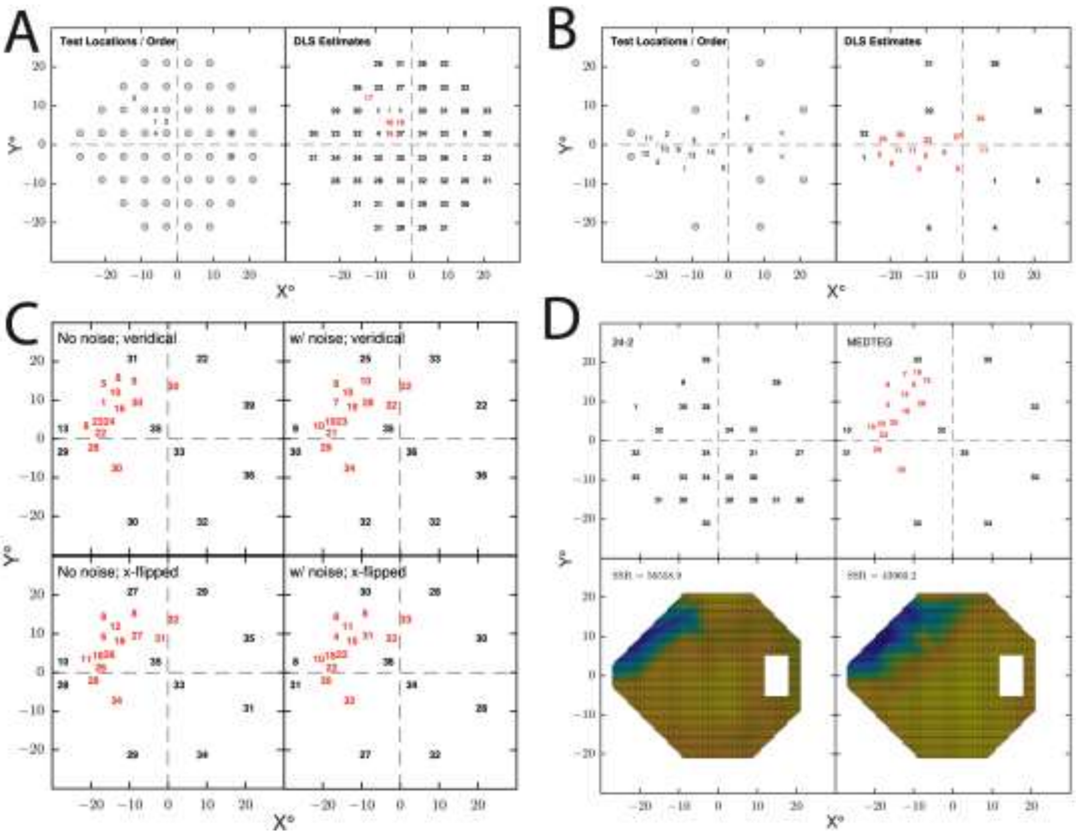

Supplemental Figure S1: Example results for each of the four simulated use cases, if, instead of computing minimum weighted entropy [$\omega E(\Delta Hdeg^2)$], one instead selected the candidate point with the greatest difference in estimated threshold between any two pairs of neighbors ("maximum gradient"). See Figures 7-11 of main manuscript for equivalent results using MEDTEG. The present results were generated by manually setting the variable "USE_MAX_DIFF_INSTEAD" to true, in the MEDTEG.m MATLAB file.
